# Cytotoxic and Antiproliferative Activity of LASSBio-2208 and the Attempts to Determine Its Drug Metabolism and Pharmacokinetics In Vitro Profile

**DOI:** 10.3390/ph17030389

**Published:** 2024-03-18

**Authors:** Raysa Magali Pillpe-Meza, Wesley Leandro Gouveia, Gisele Barbosa, Carlos A. M. Fraga, Eliezer J. Barreiro, Lidia Moreira Lima

**Affiliations:** 1 Laboratório de Avaliação e Síntese de Substâncias Bioativas (LASSBio®), Universidade Federal do Rio de Janeiro, Rio de Janeiro 21941-902, RJ, Brazil; rpillpe@gmail.com (R.M.P.-M.); wleandrogouveia@gmail.com (W.L.G.); bgiselejf@gmail.com (G.B.); cmfraga@ccsdecania.ufrj.br (C.A.M.F.); ejbarreiro@ccsdecania.ufrj.br (E.J.B.); 2 Programa de Pós-Graduação em Farmacologia e Química Medicinal, Instituto de Ciências Biomédicas, Universidade Federal do Rio de Janeiro, Rio de Janeiro 21941-902, RJ, Brazil

**Keywords:** cancer, PI3K, HDAC-6, cytotoxicity, antiproliferative activity, synergism, DMPK

## Abstract

Inappropriate expression of histone deacetylase (HDAC-6) and deregulation of the phosphatidylinositol 3-kinase (PI3K) signalling pathway are common aberrations observed in cancers. LASSBio-2208, has been previously described as a dual inhibitor in the nanomolar range of HDAC-6 and PI3Kα and is three times more potent in inhibiting HDAC-6. In this paper we described the cytotoxic and antiproliferative potency of LASSBio-2208 on different tumour cell lines, its possible synergism effect in association with PI3K and HDAC-6 inhibitors, and its drug metabolism and pharmacokinetics (DMPK) in vitro profile. Our studies have demonstrated that LASSBio-2208 has moderate cytotoxic potency on breast cancer cell line MCF-7 (IC_50_ = 23 µM), human leukaemia cell line CCRF-CEM (IC_50_ = 8.54 µM) and T lymphoblast cell line MOLT-4 (IC_50_ = 7.15 µM), with no cytotoxic effect on human peripheral blood mononuclear cells (hPBMC). In addition, it has a good antiproliferative effect on MCF-7 cells (IC_50_ = 5.44 µM), low absorption by parallel artificial membrane permeability—gastrointestinal tract (PAMPA—GIT) and low permeation by parallel artificial membrane permeability—blood–brain barrier (BBB) (PAMPA—BBB), exhibiting high metabolic stability in rat plasma. Moreover, LASSBio-2208 exhibited synergism when combined with getadolisib and tubastatin A, using the concentrations corresponding to their CC_50_ values on MOLT-4 and CCRF-CEM cells.

## 1. Introduction

Genetic mutations and aberrant epigenetic changes are the stimulus for carcinogenesis. Inadequate expression of histone deacetylases (HDACs), epigenetic modifiers, and deregulation of the signalling pathway of the phosphatidylinositol-3 kinases (PI3K) are common aberrations observed in human diseases, particularly cancers. Therefore, the simultaneous inhibition of HDACs and PI3Ks appears to exhibit synergistic therapeutic efficacy and has encouraged the development of dual inhibitors of both biochemical pathways [1,2,3,4,5].

**Figure 1:**
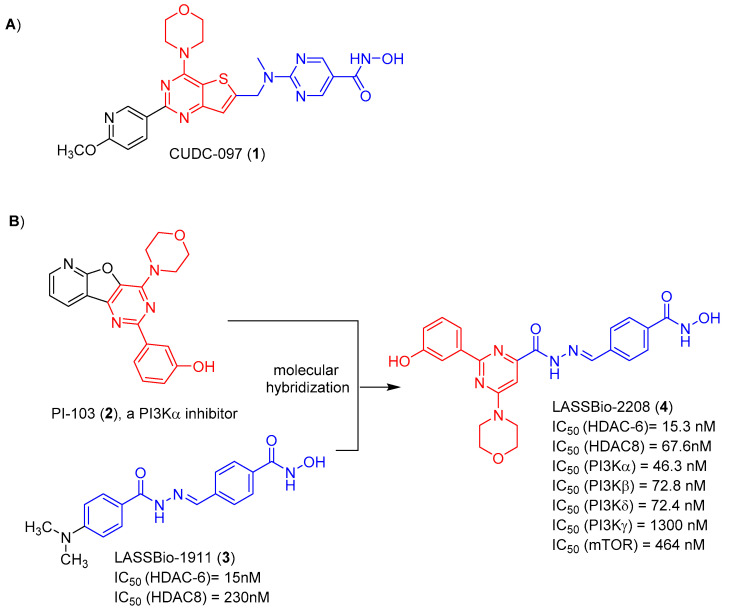
(**A**) Structure of the dual PI3K and HDACs inhibitors CUDC-097. (**B**) Design conception of LASSBio-2208 as a hybrid between PI-103 and LASSBio-1911. LASSBio-2208 and LASSBio-1911 IC_50_ values were obtained from Rodrigues et al. [7] and Rodrigues et al. [9], respectively.

Among the several hybrid compounds designed as dual inhibitors of HDAC and PI3K, CUDC-907 (**1**) has gained prominence. It is a small molecule with good oral bioavailability and is able to simultaneously inhibit PI3K and HDAC. It was designed by the introduction of a hydroxamic acid group, as the zinc-binding pharmacophore of HDAC, and the morpholinopyrimidine subunit, as the pharmacophore with which to hinge the binding of PI3K. Both are separated by an appropriate spacer, as shown in Figure 1 [6].

Considering the data in the literature that point to the potentiation of the cytotoxic effect as a result of the synergism promoted by the simultaneous inhibition of HDACs and PI3K inhibitors, we have previously described the design, synthesis and in vitro evaluation of the pharmacological profile of a series of N-arylhydrazones, planned as dual HDAC-6/8 and PI3K inhibitors [7]. The design concept of this series was based on the molecular hybridization between the morpholinopyrimidine fragment (coloured red) of PI-103 (**2**) and the acylhydrazone functionalized by a hydroxamic acid (coloured blue) framework present in LASSBio-1911 (**3**) (Figure 1). From this series, LASSBio-2208 (**4**) has stood out. This compound inhibited HDAC-6 with a potency (IC_50_) of 15.3 nM and PI3Kα with IC_50_ of 46.3 nM. It also inhibited PI3Kβ, PI3Kδ and HDAC8 with an IC_50_ of 72.8 nM, 72.4 nM and 67.6 nM, respectively [7] (Figure 1). This lead compound displayed good lipophilicity, with a calculated logP value of 1.96, and low aqueous solubility (1.81 μM) [7]. The cytotoxic effect of LASSBio-2208 (**4**) has been partially studied and published by Guerra et al. [8], using the PC3 prostate carcinoma cell line. The tests were carried out using the 24 h and 48 h MTT method. It was observed that, after 24 h of treatment with LASSBio-2208 (**4**) at concentrations of 0.01 µM, 0.05 µM, 0.1 µM, 0.5 µM, 1 µM and 5 µM, cell viability was reduced by almost 50% at the highest concentration studied. However, after 48 h of treatment, at the same concentrations, there was no effect on cell viability. Its cytotoxic potency has not been determined. Next, the authors observed, through flow cytometry assays, that LASSBio-2208 (**4**) led to initial cell apoptosis in 60–66% of the cell population and favoured cell cycle arrest in the G2/M phase, in the 24 h assay [8].

In this paper, we describe the cytotoxic potency of LASSBio-2208 (**4**) in different tumour cells lines, its antiproliferative profile, the study of pharmacological synergism between LASSBio-2208 (**4**) with gedatolisib (PI3K and mTOR inhibitor), tubastatin A (HDAC-6 inhibitor), or LASSBio-1911 (**3**) (HDAC-6/8 inhibitor), and the attempts to determine its in vitro drug metabolism and pharmacokinetics (DMPK) profile.

## 2. Results and Discussion

### 2.1. Pharmacological Experiments

#### 2.1.1. Cell Viability by MTT and CC_50_ Determination

To determine the cytotoxic effect of LASSBio-2208 (**4**), using the MTT assay, four human cell lines with mutations in the PI3K pathway were selected: human prostate cancer cell line (PC-3), T lymphoblast cell line (MOLT-4), human leukaemia cell line (CCRF-CEM) and breast cancer cell line (MCF-7). Three controls were used in our experiments: the dual PI3K and mTOR inhibitor gedatolisib (IC_50_ (PI3Kα) = 0.4 nM, IC_50_ (PI3Kβ) = 6 nM, IC_50_ (PI3Kδ) = 6 nM, IC_50_ (PI3Kγ) = 5.4 nM and IC_50_ (mTOR) = 1.6 nM) [10]; the HDAC-6 inhibitor tubastatin A (IC_50_ = 15 nM) [11]; and the HDAC-6/8 inhibitor LASSBio-1911. The MTT studies were performed at 24 h, 48 h and 72 h.

As demonstrated in Table 1, gedatolisib displayed a time-dependent cytotoxic effect on all four tumour cell lines studied. The best results were found in the 48 h MTT and were maintained in the 72 h MTT assay. Considering the cell viability studies in the MTT of 72 h, gedatolisib displayed average cytotoxic concentration (CC_50_) values of 0.86 µM, 70 nM, 90 nM and 60 nM to PC-3, MOLT-4, CCRF-CEM and MCF-7, respectively. These results are similar to the values of 0.54 µM, 20 nM, 30 nM e 70 nM, respectively, previously described by Costa et al. [12] in regard to the performance of gedatolisib against the same tumour cell lines (Table 1). It is worth noting that our work is the first to describe the cytotoxic effect of gedatolisib in 24 h and 48 h MTT assays on PC-3 and MCF-7 tumour cell lines.

Unlike gedatolisib (a classical pan inhibitor of PI3K and mTOR), tubastatin A (a selective inhibitor of HDAC-6) did not show any cytotoxic activity in the 24 h MTT assay on solid tumour cell lines. At longer times (48 h and 72 h) it showed low cytotoxic potency on the PC3 and MCF7 lines, ranging from 34.89 to 65.94 μM for PC3 and 83.56 to >100 μM for MCF-7 (Table 1). However, it proved to be equipotent in inhibiting leukaemia strains (CCRF-CEM and MOLT-4), exhibiting CC_50_ varying from 5.65 μM to 12.51 μM.

The third standard used in our experiments was the compound LASSBio-1911, described as an inhibitor of HDAC-6 and HDAC8. As expected, this inhibitor had a cytotoxic action profile like tubastatin A, showing greater cytotoxic potency on human leukaemia cell lines, with CC_50_ ranging from 5.27 μM to 13.75 μM (Table 1).

Having studied the cytotoxic response of the standards, gedatolisib, tubastatin A and LASSBio-1911, on the two solid tumour cell lines and two selected leukaemia cell lines, we began studies to determine the cytotoxic potency of LASSBio-2208. As demonstrated in Table 1, LASSBio-2208 was inactive in the PC3 strain in the 24 h, 48 h and 72 h MTT assays. However, it showed a CC_50_ of 31.71 μM and 23.0 μM on MCF-7 cells in the 48 h and 72 h MTT assays, respectively, although with a low maximum cytotoxic response (E_max_ of 57.3% and 60.7%, respectively).

LASSBio-2208 cytotoxic effect against leukaemia cell lines was significantly better. On MOLT-4 cells it exhibited a CC_50_ of less than 10 μM at all times studied. As shown in Table 1, it exhibited a CC_50_ of 7.14 μM, 8.26 μM and 7.15 μM on MOLT-4 in the MTT assays at 24 h, 48 h and 72 h, respectively. Good results were also found on CCRF-CEM, exhibiting CC_50_ of 27.75 μM (24 h), 8.68 μM (48 h) and 8.54 μM (72 h).

The results obtained with LASSBio-2208 reveal a cytotoxic action profile in a phenotypic model very similar to tubastatin A, but different from gedatolisib. Unlike gedatolisib, the cytotoxic effect of LASSBio-2208 does not appear to be time-dependent and exhibits little or no cytotoxic effect on selected solid tumour cell lines, which overexpress the PI3K pathway (PTEN-deficient PC3 and MCF7 with *PIK3CA* mutation) [13,14]. Considering that LASSBio-2208 is preferentially a dual inhibitor of PI3Kα and HDAC-6, these results can be considered unexpected. However, it must be considered that LASSBio-2208 is three times more potent at inhibiting HDAC-6 (IC_50_ = 15.3 nM) than at inhibiting PI3Kα (IC_50_ = 46.3 nM) [7]. These data, together with its very low aqueous solubility (thermodynamic solubility = 1.81 μM) [7], may indicate that, under the conditions of the experiment, the soluble fraction of LASSBio-2208 could reach concentrations at which it would only or preferentially inhibit HDAC-6.

The observation of a time-independent cytotoxic effect for tubastatin A and LASSBio-2208 may at first seem unexpected, but it is not. Histone deacetylases (HDACs) are epigenetic modifiers, but HDAC-6 has a greater presence in the cytosol of the cell, being found in smaller quantities in the cell nucleus [15,16,17]. Therefore, the cellular responses mediated by HDAC-6 and its inhibitors are mostly non-genomic.

#### 2.1.2. Determination of Selectivity Index (SI) by MTT Assay

To determine the cytotoxic selectivity index of LASSBio-2208 and the standards tubastatin A and LASSBio-1911, we performed a 48 h MTT assay using human peripheral blood mononuclear cells (hPBMC), as a model of non-tumour cells. The SI indicates how selective a compound is for a tumour cell line compared with a non-tumour cell line [18].

Both LASSBio-1911 and LASSBio-2208, even at the highest concentration tested (100 µM), showed no cytotoxic effect on hPBMC. At this concentration (100 µM) they showed a maximum cytotoxic response of 31.1% and 29.9%, respectively; therefore, the value of their CC_50_ cannot be determined (Table 2). Concentrations higher than 100 μM were not tested due to the insolubility of LASSBio-2208 in the culture medium. Comparatively, tubastatin A proved to be more cytotoxic to human non-tumour cells, exhibiting a CC_50_ of 85.66 μM and a maximum cytotoxic response (Emax) of 58.2% (Table 2).

Table 2 shows the comparative results of the cytotoxic effect (measured in a 48 h MTT test) for LASSBio-1911, tubastatin A and LASSBio-2208 on hPBMC and human tumour cell lines (PC3, MCF-7, MOLT-4 and CCRF-CEM). The cytotoxic selectivity index was calculated by the ratio of CC_50_ on non-tumour cells to CC_50_ on tumour cells [18].

Tubastatin A showed a cytotoxic selectivity index (SI) of 11.4 and 9.1 for MOLT-4 and CCRF-CEM, which means that it needs a concentration 11.4 and 9.1 times higher to kill the non-tumour cells (hPBMC) than the tumour cell lines MOLT-4 and CCRF-CEM, respectively. LASSBio-1911 and LASSBio-2208 did not have their SI calculated, since it was not possible to determine their CC_50_ on hPBMC.

#### 2.1.3. Cell Viability and Antiproliferative Profile by SRB Assay

Considering the limitations of the MTT assay [19], we decided to investigate LASSBio-2208 antiproliferative profile against the PC-3, MCF-7, CCRF-CEM and MOLT-4 cell lines, using the sulforhodamine B (SRB) assay at 48 h. For comparison purposes, the standards gedatolisib, tubastatin A and LASSBio-1911 were subjected to the same tests.

As can be seen by comparing the results shown in Table 1 and Table 3, all the compounds are much less antiproliferative than they are cytotoxic. In PC-3 cells, none of the compounds showed a maximum antiproliferative response (Emax) of more than 60%, when tested at the highest concentration of 100 μM. The CC_50_ for LASSBio-1911 and tubastatin A in the SRB assay on the PC-3 was not calculated, as they displayed a maximum response inferior to 35% at 100 μM. Gedatolisib and LASSBio-2208, which exhibited maximum response greater than 50% at a concentration of 100 μM, were selected for determination of their CC_50_. As demonstrated in Table 3, LASSBio-2208 showed an antiproliferative potency 16 times lower than gedatolisib (CC_50_ = 98.93 μM versus 6.16 μM, respectively) (Table 3).

When the test was carried out with the second human solid tumour cell line MCF-7, very different behaviour was observed. Tubastatin A and LASSBio-1911 displayed a CC_50_ of 66.01 µM and 39.14 µM, respectively, and a maximum effect of over 70% for the first and over 80% for the second. LASSBio-2208 was 7 to 12 times more potent than LASSBio-1911 and tubastatin A, respectively. However, it was 181 times less potent than gedatolisib, which showed a CC_50_ of 0.03 µM (Table 3).

Surprisingly, the antiproliferative effect of LASSBio-2208 on human leukaemia cell lines (CCRF-CEM and MOLT-4) was null. This inactivity cannot be correlated to the compound’s mechanism of action, given that the standards gedatolisib (PI3K inhibitor), tubastatin A (HDAC-6 inhibitor) and LASSBio-1911 (HDAC-6/8 inhibitor) exhibited antiproliferative effect with CC_50_ of 0.78 µM, 9.69 µM and 25.18 µM (respectively) on CCRF-CEM, and CC_50_ of 3.68 µM, 10.45 µM and 53.09 µM (respectively) on MOLT 4 (Table 3).

#### 2.1.4. Synergism Study by MTT on CCRF-CEM Cell Line

To investigate the synergistic effect of LASSBio-2208 with inhibitors of PI3K (gedatolisib), HDAC-6 (tubastatin A) and HDAC-6/8 (LASSBio-1911) we carried out the 48 h MTT test, using different combinations of concentrations of the target compounds. The proportions of combinations (based on the CC_50_ values obtained for each target compound) were established in the 48 h MTT assay on CCRF-CEM cell lines. The combination indices (CI) were obtained using the COMPUSYN software (https://www.combosyn.com/register.html, accessed on 10 March 2024), which was developed to allow the analysis of combinations of compounds that act by modulating different targets or pathways [20], which were in turn classified according to the parameters indicated in the literature as synergism, addition and/or antagonism [21].

The first combination evaluated was an association of gedatolisib and LASSBio-1911. We observed that, in combinations below the CC_50_ value, moderate antagonistic effects were detected. However, as the proportional combination of CC_50_ was increased, a synergistic effect was detected between them (Table 4).

The second combination investigated was between gedatolisib and tubastatin A, for which we observed additive effects in combinations using lower concentrations of the compounds, while there was a prevalence of the synergistic effect as the concentrations increased (Table 5). Our results agree with literature data [22,23], which describe that the combination of PI3K and HDAC-6 inhibitors has synergistic effects, resulting in an increase in the cytotoxic activity of the combination when compared with the cytotoxic effect of the species alone, as we have found for gedatolisib and tubastatin A (Table 4).

LASSBio-2208, previously described as an inhibitor of PI3Kα (IC_50_ = 46.3 nM), PI3Kβ (IC_50_ = 72.8 nM), PI3Kδ (IC_50_ = 72.4 nM) and HDAC-6 (IC_50_ = 15.3 nM) (Figure 1), displayed both synergistic and additive effects, when combined with gedatolisib and tubastatin A. As demonstrated in Table 4, the combination of LASSBio-2208 using its CC_50_ value and that calculated for gedatolisib or tubastatin A resulted in a synergic response. A strong synergism was observed when combining LASSBio-2208 and getadolisib at concentrations four times higher than their CC_50_ values (Table 4). On the other hand, the combination using concentrations four times higher than the CC_50_ values of LASSBio-2208 and tubastatin A resulted in an additive effect. Using this same combination of concentrations (4 × CC_50_ values), the combination of the pairs LASSBio-2208 and LASSBio-1911 and getatolisib with tubastatin A, resulted in a nearly additive effect (Table 4).

#### 2.1.5. Synergism Study by MTT on MOLT-4 Cell Line

Continuing the synergism studies, trials were carried out with the second acute lymphoblastic leukaemia strain, MOLT-4. Combination of gedatolisib and tubastatin A showed a synergistic effect when the individual concentrations were set at values ranging from 0.25 to 2 times the value of their CC_50_. However, antagonism was observed when both standard drugs were combined using concentrations four times higher than their CC_50_ values (Table 5). At the highest combined concentration (4 × CC_50_ value), gedatolisib also had an additive effect when combined with LASSBio-1911, and a synergistic effect when combined with LASSBio-2208. Regarding LASSBio-2208, it exhibited synergism when combined with all of the standards studied (gedatolisib, LASSBio-1911 and tubastatin A), using the concentration corresponding to their CC_50_ values on MOLT-4 cells or values two times higher than their CC_50_ (Table 5). Its combination with tubastatin A using 1/4 of the concentration estimated for its CC_50_ or 4 times greater than the CC_50_, resulted in the indication of a pharmacological antagonism between LASSBio-2208 and tubastatin A. Similar results were found for the combination of LASSBio-2208 with LASSBio-1911, at concentrations 1/4 of the CC_50_ or 4 times higher than the CC_50_.

### 2.2. In Vitro DMPK Studies

#### 2.2.1. Parallel Artificial Membrane Permeability Assay (PAMPA)

To obtain information on the permeability potential of LASSBio-2208 on cell membranes, this derivative was tested using the parallel artificial membrane permeability assay (PAMPA) that is used as an in vitro model of passive diffusion and transcellular permeation [24,25]. In this model, the permeability across two artificial membranes is measured: brain lipid from pig extract in dodecane (called PAMPA—BBB) and L-α soy phosphatidylcholine in dodecane (called PAMPA—TGI). The experiments were carried out in comparison with the gedatolisib, tubastatin A and LASSBio-1911 standards (Table 6).

As demonstrated in Table 6, gedatolisib was insoluble under the experimental conditions used to determine permeability in PAMPA—BBB. Unlike tubastatin A, which was shown to be able to permeate the artificial membrane that mimics the blood–brain barrier and was therefore predicted to be able to permeate it (CNS+); LASSBio-2208 and LASSBio-1911 showed low permeation on PAMPA—BBB (Table 6). Therefore. they were predicted to be unable to permeate the blood–brain barrier (CNS−).

The permeation profile of the four compounds in PAMPA—GIT was somewhat different. LASSBio-2208 and LASSBio-1911 exhibited a low probability of absorption by the gastrointestinal tract, with Pe = 0.39 × 10^−6^ cm/s and Pe = 0.3592 × 10^−6^ and fractions absorbed of 13.78% and 20.15%, respectively. Gedatolisib and tubastatin A displayed medium probability of absorption by the gastrointestinal tract with Pe = 1.74 × 10^−6^ cm/s and Pe = 1.982 × 10^−6^ and fractions absorbed of 48.4% and 52.9%, respectively. The permeation profile found for LASSBio-2208 is consistent with its low logP value (1.96) and low aqueous solubility (1.81 μM) described by Rodrigues et al. in 2020 [7].

#### 2.2.2. Metabolic Stability

Further, the metabolic stability of LASSBio-2208 was studied using rat plasma and rat liver microsomes (RLM).

Considering that both LASSBio-2208 and LASSBio-1911 have, in their structures, the *N*-acylhydrazone and hydroxamic acid fragments, both susceptible to metabolic hydrolysis [26,27,28], metabolic stability in plasma was conducted with both compounds. As demonstrated in Table 7, both compounds showed a low rate of metabolization and high half-life, indicating high metabolic stability in rat plasma.

However, attempts to study the metabolic stability of LASSBio-2208 in rat liver microsomes were all unsuccessful. Although it was possible to visualize the disappearance of the signal corresponding to LASSBio-2208 in the chromatograms from different times of the metabolic stability experiment in RLM, it was not possible to observe the emergence of any new signal, characteristic of metabolite formation. This atypical pattern could be associated with the ability of hydroxamic acid to exhibit strong cation chelating properties, including with the iron atom of the CYP450 proteins [29,30]. Another hypothesis to explain the results is linked to the potential ability of LASSBio-2208 to form nonspecific bonds to membrane lipid proteins of a microsomal fraction. Once such bonds are formed, the bound fraction would become incapable of being metabolized. To corroborate or refute this hypothesis, we carried out a nonspecific microsome binding assay [31] with LASSBio-2208, and with the standards, gedatolisib, tubastatin A and LASSBio-1911.

As exemplified in Table 8, the methodology was validated using tolbutamide as a standard. In our experiments we found an unbound fraction (*ƒ_u_*_(mic)_) in the nonspecific microsomal binding for tolbutamine of 1.113, similar to the value previously described in the literature by McLure et al. [31]. Then, the unbound fraction in the nonspecific microsomal binding assay for compounds LASSBio-2208, LASSBio-1911, gedatolisib and tubastatin A were determined. The extent of free drug, i.e., not bound to microsomal membrane, is depicted in Table 8. In general, the more lipid soluble a compound is, the more the lipid proteins can be bound to a membrane. In our results, tubastatin A and LASSBio-1911 bound extensively to microsomal membrane, exhibiting a *ƒ_u_*_(mic)_ = 0.131 and 0.004, respectively. While gedatolisib showed a medium binding rate (*ƒ_u_*_(mic)_ = 0.503). On the other hand, LASSBio-2208 exhibited the smaller extension of nonspecific microsomal binding (*ƒ_u_*_(mic)_ = 0.937), with more than 93% unbounded and, therefore, available to be metabolized. Taken together, these results refute the hypothesis that there was no metabolic conversion of LASSBio-2208 in RLM due to possible non-specific binding to microsomal proteins, resulting in a low fraction of free drug under the experimental conditions. Studies to investigate the potential of LASSBio-2208 to inhibit CYP isoforms and therefore prevent its own metabolism need to be carried out further.

## 3. Materials and Methods

### 3.1. Cell Culture

The tumour cell lines used in this study were acute lymphoblastic leukaemia with mutation in PTEN (CCRF-CEM), acute lymphoblastic leukaemia with mutation in PTEN and PI3KR1 (MOLT-4), breast cancer with mutation in PI3KCA (MCF-7) and prostate cancer with mutation in PTEN (PC-3), these cells were thawed and maintained in RPMI-1640 medium supplemented with 10% foetal bovine serum (FBS), with 1% penicillin antibiotic (100 U/mL) and streptomycin (100 µg/mL) in an oven at 37 °C and 5% CO_2_. The concentration of the compounds used for the tests ranged from 0.003–100 µM with DMSO at 1%, except for gedatolisib where the maximum concentration evaluated was 50 µM.

Human peripheral blood mononuclear cells (PBMCs) were used to determine the selectivity index (SI) and were obtained from healthy volunteers under a protocol approved by the Ethics Committee CAAE: 38257914.7.0000.5259. Immortalized cells were also obtained from the Rio de Janeiro Cell Bank (BCRJ).

### 3.2. Compounds and LC/UV Analysis

The gedatolisib and tubastatin A standards were purchased commercially from Sigma-Aldrich (Merck, Rahway, NJ, USA) with product codes PZ0281 and SML0044, respectively. LASSBio-1911 and LASSBio-2208 were obtained from the LASSBio chemistry library. The compounds were first analysed by high-performance liquid chromatography (HPLC) using a Shimadzu Prominence system (Shimadzu, Tokyo, Japan), consisting of a vacuum degasser (DGU-20A5), a binary pump (LC-20AD), an automatic sampler (SIL-20A), a UV/VIS photodiode array detector (SPD-M20A) and a C8 column (Kromasil C8 250 mm 4.6 mm, 5 µM), operating at room temperature. An isocratic mobile phase was used for each compound. For gedatolisib and LASSBio-1911 the mobile phase consisted of acetonitrile:acidified water (90:10 *v*/*v*). For tubastatin A and LASSBio-2208, acetonitrile:acidified water (50:50 *v*/*v*) was used. Both analyses were conducted at a flow rate of 1 mL/min (Figures S1–S4).

### 3.3. Cell Viability Assay by MTT

The cells were plated in a 96-well plate (3 × 10^4^ cell/well), after which the test compounds were added at the concentrations mentioned above to be incubated for 24 h, 48 h and 72 h in an oven at 37 °C and 5% CO_2_. After the incubation time, the plate was centrifuged (Universal centrifuge 320R Hettich, Kirchlengern, Germany) at 440× *g*, 10 min and 4 °C, then 110 µL of supernatant was removed and 10 µL of MTT solution was added and left to incubate for 3.5 h in the oven at 37 °C and 5% CO_2_. Finally, to dissolve the formazan crystals, 100 µL of sodium dodecyl sulphate-hydrochloric acid (SDS–HCl) detergent was added and left to dissolve until the following day to be read on the Molecular Devices Spectramax M5 plate reader at a wavelength of 595 nm. The CC_50_ was calculated using the GraphPad Prism 9.0 program. Each sample was tested in triplicate in three independent experiments (N3) [32,33].

### 3.4. PBMC Viability Assay by MTT

First, 8 mL of blood from each healthy donor (3 people) was collected in a tube with EDTA in a 1:20 ratio to prevent the blood from clotting. In a 15 mL Falcon tube containing 4 mL of Ficoll-Paque, the 8 mL of blood collected was slowly added, letting it fall down the walls of the tube. It was centrifuged (Universal Centrifuge 320R Hettich) for 40 min at 750× *g* at 21 °C with acceleration 1 and brake 0. After centrifugation, in order to extract the ring of mononuclear cells, the supernatant above it was first carefully discarded so as not to disturb the ring. The ring was then slowly removed and placed in another 15 mL Falcon tube to which pure RPMI-1640 medium was added in a 1:1 ratio and centrifuged for 10 min at 580× *g* and 21 °C with acceleration 8 and brake 9 [34,35].

The supernatant was discarded, and the pellet was resuspended in 1 mL of RPMI-1640 medium supplemented with 10% SBF and 1% antibiotic. An amount of 20 µL of cells were taken from the mixture and added to 380 µL of trypan blue dye for counting in the Neubauer chamber. Considering that 80% of PBMCs are lymphocytes and the rest are monocytes [36], the cells were plated with the aim of having 10^5^ cell/well. The 96-well plate was left in the oven at 37 °C and 5% CO_2_ for 1 h, so that the monocytes adhered to the bottom of the plate; after this time, the supernatant containing the lymphocytes was transferred to another plate and the protocol established for the MTT assay indicated in Section 3.2 was continued.

### 3.5. Cell Viability Assay by SRB

The cells were first plated in a 96-well plate (3 × 10^4^ cell/well) and incubated in the oven at 37 °C and 5% CO_2_ for 24 h. The test compounds were then added at the concentrations indicated in Section 3.1 and the plate was incubated again for 48 h. After 48 h, in order to fix the cells, they were centrifuged (Universal Centrifuge 320R Hettich) at 440× *g* for 10 min at 4 °C and trichloroacetic acid (TCA) was added. For adherent cells, all the supernatant was removed and 100 µL of 10% TCA was added to each well, keeping the plate in the fridge at 4 °C for 1 h. For the cells in suspension, without removing the supernatant, 50 µL of 80% TCA was added to each well and the plate kept in the refrigerator at 4 °C for 2 h [37,38].

After the fixation time, the TCA was discarded in the sink and the wells washed four times with distilled water. The plate was dried at room temperature and then stained with 100 µL of sulforhodamine B (SRB) at 0.057% m/v solubilized in 1% acetic acid, and the plate was left to stand in the dark for 30 min. After this time, the excess SRB was discarded and washed with 1% acetic acid (4 times of 15 mL) [38,39].

After drying at room temperature, the dry plate was taken to the Spectramax M5 plate reader from Molecular Devices in order to read the absorbance at 510 nm. Before reading, the SRB had to be dissolved in a Tris-base solution (pH 10.5). In this way, 100 µL of the solution was added to each well and the plate was gently shaken for 15 min. Once this time had elapsed, the readings were taken. The results obtained were analysed using GraphPad Prism 9.0.

### 3.6. Synergism Assay by MTT

The MTT test was used to evaluate synergism between the target compounds, adapting the experimental procedures already described in Section 3.2. This was done in order to include the choice of compound concentrations, as indicated by the developers of the COMPUSYN software. To determine the concentrations used in the experiment, it was first necessary to determine the CC_50_ of the compounds. Once this stage had been completed, tests were carried out on combinations with concentrations proportional to the CC_50_ for each target compound (i.e., 0.25 × CC_50_, 0.5 × CC_50_, 1 × CC_50_, 2 × CC_50_, 4 × CC_50_) [20,21].

### 3.7. Parallel Artificial Membrane Permeability Assay (PAMPA)

To perform the permeability assay we used a donor plate where the compounds (tests or controls) are diluted in a buffered medium, characterized by the presence of a synthetic membrane of polyvinylidene fluoride (PVDF) impregnated with a lipid solution, forming a barrier through which the compounds migrate through a diffusion process to the lower plate called the receptor.

The lipid mixture that impregnates the filter has a different constitution for the permeability tests for the blood–brain barrier (brain lipid from pig extract in dodecane) and gastrointestinal tract (L-α soy phosphatidylcholine in dodecane). In the PAMPA—BBB and PAMPA—GIT assays, the optical density values obtained in the reading at each selected wavelength, for each of the compounds were analysed in comparison with the values of several controls. In the case of the BBB, these values were used to elaborate an equation and determine the permeability coefficient (Pe), using a previously elaborated spreadsheet, and, for the PAMPA—GIT, these values were used to determine the absorbed fraction (Fa%), both using the Excel program. The permeability result for PAMPA—GIT classifies the compounds according to the percentage of absorbed fraction (Fa%), as follows: high intestinal permeability (70–100%), medium permeability (30–69%) or low permeability (0–29%), and the samples were diluted from a stock solution of 10 mM. The PAMPA—BBB model classifies the compounds only as follows: permeable (CNS+) or non-permeable (CNS−) and the assays were made from a stock solution of 1 mg for each compound [25,40,41,42].

### 3.8. Plasma Stability

LASSBio-2208 and LASSBio-1911, in a final concentration of 10 μM from stock solution of 1 mM, was added in 50 μL of rat plasma solution diluted in 250 μL with phosphate buffer (pH 7.4) and was placed in a shaker at 37 °C under vigorous stirring for 0, 30, 60, 120 and 240 min. After each reaction time, 1 mL of cold acetonitrile containing 2 uM internal standard was added to the wells to stop the reaction. The solution was mixed and centrifuged at 24,500× *g* for 15 min (Universal Centrifuge 320R Hettich) [43,44]. The supernatant (1 mL) was filtrated and placed in vials to be analysed by HPLC.

### 3.9. Microsomal Stability

To evaluate microsomal stability, we added 10 µM of a 1 mM stock solution of LASSBio-2208 and LASSBio-1911 to a mixture containing rat liver microsomes at a concentration of 1 mg/mL of proteins. This mixture was supplemented with the essential cofactors for activating the NADPH-generating system, including 1.3 mM MgCl2, 0.4 mM NADP+, 3.5 mM glucose 6-phosphate, and 0.5 U/mL glucose 6-phosphate dehydrogenase, all diluted in 0.1 M phosphate buffer (pH 7.4) to a final volume of 250 µL [45].

The samples were then subjected to a pre-incubation at 37 °C, followed by incubation at the same temperature with constant agitation for varying time intervals (0, 15, 30, 45, 60 min). Subsequently, the reaction was halted by adding 1 mL of an acetonitrile:methanol solution (1:1) containing 2 µM of the internal standard, enabling the extraction of compounds and precipitation of proteins. The samples were then centrifuged at 24,500× *g* for 15 min at 4 °C to separate the components of the mixture. The resulting supernatant (1 mL) was carefully collected, filtered, and subsequently analysed by HPLC. This procedure was conducted both in the presence and absence of the enzyme cofactors and replicated in triplicate [45].

### 3.10. Non-Specific Microsome Protein Binding

Liver microsomes were diluted with PBS pH 7.4 to a concentration of 0.5 mg protein/mL and 3 µL at 1 mM of the working test compound and solutions were mixed with 357 µL of liver microsome suspension. After mixing, duplicate 200 µL aliquots were immediately removed, while another microtubule acted as the control sample (T 0 h), and were quenched with 400 µL of cold acetonitrile containing analytical internal standards. The other samples were incubated at 37 °C at 100 rpm, for 60 min with the same subsequent treatment. After this, all samples were centrifuged at 3220× *g* for 30 min at room temperature, the supernatant was collected and analysed by HPLC. The results were obtained by peak area ratio, which was derived via normalizing to an analytical internal standard, and the unbound fraction (*f_u_*_(mic)_) of drug in a microsomal compartment was expressed as the free drug concentration (peak area in time 0 min) divided by the total drug concentration (peak area in time 60 min) [46,47].

## 4. Conclusions

Our results strongly indicate that LASSBio-2208, despite its potency in the nanomolar range on the PI3K and HDAC-6 targets (Figure 1), has inadequate physicochemical properties, reflecting a low permeation and absorption profile, with impacts on its cytotoxic and antiproliferative potency on human solid tumour and leukaemia cell lines. Despite its good plasma stability, the failure to determine its metabolic stability in RLM may be related to the ability of this derivative to inhibit isoforms of the CYP system, which should be investigated in the future. Our data also point to the possibility of a synergistic effect, by increasing LASSBio-2208 cytotoxic activity if combined with gedatolisib or tubastatin A. Taken together, our data indicate the need to optimize LASSBio-2208 for subsequent proof-of-concept studies in in vivo models. Therefore, further studies should be conducted to optimize the pharmacodynamic and pharmacokinetic properties of this important lead compound.

## Figures and Tables

**Table 1:** CC_50_ values of the standards, gedatolisib, tubastatin A and LASSBio-1911, in the MTT viability assay at 24 h, 48 h and 72 h.

MTT Assay	Cell Line	Gedatolisib CC_50_ (µM)	Tubastatin A CC_50_ (µM)	LASSBio-1911 CC_50_ (µM)	LASSBio-2208 CC_50_ (µM)
24 h	PC-3	>100 ^a^ E_max_ = 48%	>100 ^a^ E_max_ = 41.8%	>100 ^a^ E_max_ = 26.4%	>100 ^a^ E_max_ = 18.4%
	MCF-7	5.04 (2.86–8.88) E_max_ = 79.9%	>100 ^a^ E_max_ = 40.1%	>100 ^a^ E_max_ = 21.6%	>100 ^a^ E_max_ = 29.5%
	CCRF-CEM	0.31 (0.24–0.41) E_max_ = 98.6%	12.51 (5.95–26.31) E_max_ = 95.8%	9.69 (8.07–11.64) E_max_ = 98%	27.75 (21.70–35.50) E_max_ = 69.4%
	MOLT-4	0.35 (0.25–0.47) E_max_ = 97.2%	5.67 (3.95–8.12) E_max_ = 94.6%	13.75 (2.55–74.05) E_max_ = 96.5%	7.14 (3.51–14.54) E_max_ = 84.1%
48 h	PC-3	5.05 (2.59–9.82) E_max_ = 60.5%	65.94 (57.29–75.88) E_max_ = 67.8%	>100 ^a^ E_max_ = 42.6%	>100 ^a^ E_max_ = 39.3%
	MCF-7	0.21 (0.13–0.34) E_max_ = 96.8%	>100 ^a^ E_max_ = 48.2%	42.62 (30.76–59.05) E_max_ = 65.4%	31.71 (19.45–51.69) E_max_ = 57.3%
	CCRF-CEM	0.085 (0.07–0.11) E_max_ = 99.5%	9.38 (5.18–17.03) E_max_ = 99.1%	6.98 (4.28–11.37) E_max_ = 98.8%	8.68 (6.99–10.30) E_max_ = 91.7%
	MOLT-4	0.08 (0.07–0.11) E_max_ = 99.3%	7.50 (5.79–9.70) E_max_ = 98.2%	6.15 (4.24–8.90) E_max_ = 98.9%	8.26 (5.82–11.71) E_max_ = 83.2%
72 h	PC-3	0.86 (0.59–1.25) E_max_ = 81.1%	34.89 (30.83–39.47) Emáx = 83.6%	77.10 (63.96–92.95) E_max_ = 59.2%	>100 ^a^ E_max_ = 42.1%
	MCF-7	0.06 (0.04–0.08) E_max_ = 99.3%	83.56 (70.22–99.42) E_max_ = 56.8%	38.43 (31.37–47.06) E_max_ = 75.4%	23.0 (15.50–34.14) E_max_ = 60.7%
	CCRF-CEM	0.09 (0.08–0.11) E_max_ = 100%	7.11 (3.32–15.19) E_max_ = 99.3%	6.92 (2.97–16.08) E_max_ = 97.1%	8.54 (6.30–11.57) E_max_ = 90.4%
	MOLT-4	0.07 (0.06–0.08) E_max_ = 99.8%	5.65 (3.51–9.09) E_max_ = 98.7%	5.27 (3.45–8.03) E_max_ = 99.2%	7.15 (5.20–9.81) E_max_ = 91.2%

Data expressed from 3 independent tests (*n* = 3), with a 95% confidence interval which is presented between “()”. E_max_ represents the maximum effect of the compound at its maximum concentration used. ^a^ CC_50_ value is estimated because, at the highest concentration of 100 μM, the compound showed a cytotoxic effect of less than 50%.

**Table 2:** CC_50_ values and selectivity index (SI) of LASSBio-1911, tubastatin A and LASSBio-2208 on non-tumour cell hPBMC and on human tumour cell lines PC-3, MCF-7, MOLT-4 and CCRF-CEM, determined in the 48 h MTT assay.

Cell Line	LASSBio-1911		Tubastatin A		LASSBio-2208	
	CC_50_ (µM) 48 h	SI	CC_50_ (µM) 48 h	SI	CC_50_ (µM) 48 h	SI
Human PBMC	>100 ^a^ E_max_ = 31.1%	N.A.	85.66 (39.7–184.8) E_max_ = 58.2%	N.A.	>100 ^a^ E_max_ = 29.9%	N.A.
PC-3	>100 E_max_ = 42.6%	N.D.	65.94 (57.29–75.88) E_max_ = 67.8%	1.29	>100 ^a^ E_max_ = 39.3%	N.D.
MCF-7	42.62 (30.76–59.05) E_max_ = 65.4%	N.D.	>100 ^a^ E_max_ = 48.2%	N.D.	31.71 (19.45–51.69) E_max_ = 57.3%	N.D.
MOLT-4	6.15 (4.24–8.90) E_max_ = 98.9%	N.D.	7.50 (5.79–9.70) E_max_ = 98.2%	11.42	8.26 (5.82–11.71) E_max_ = 83.2%	N.D.
CCRF-CEM	6.98 (4.28–11.37) E_max_ = 98.8%	N.D.	9.38 (5.18–17.03) E_max_ = 99.1%	9.13	8.68 (6.99–10.30) E_max_ = 91.7%	N.D.

Data expressed from 3 independent tests (*n* = 3), with a 95% confidence interval which is presented between “()”. E_max_ represents the maximum effect of the compound at its maximum concentration used. SI.: selectivity index, N.A.: not applicable, N.D.: not determined. ^a^ CC_50_ value was estimated, as, at the highest concentration of 100 μM, the compound showed a cytotoxic effect of less than 50%.

**Table 3:** CC_50_ values calculated from cell viability tests on immortalized PC3, MCF-7, CCRF-CEM and MOLT-4 cell lines using the SRB assay (48 h incubation).

Cell Line	Gedatolisib CC_50_ (µM)	LASSBio-1911 CC_50_ (µM)	Tubastatin A CC_50_ (µM)	LASSBio-2208 CC_50_ (µM)
PC-3	6.16 (1.42–26.7) E_max_ = 56.2%	>100 E_max_ = 34.04%	>100 E_max_ = 39.3%	98.93 (85.02–115.1) E_max_ = 51.4%
MCF-7	0.03 (0.01–0.06) E_max_ = 95.7%	39.14 (29.36–52.17) E_max_ = 86.9%	66.01 (57.45–75.95) E_max_ = 71.2%	5.44 (3.15–9.41) E_max_ = 67.4%
MOLT-4	3.68 (2.04–6.62) E_max_ = 69.2%	53.09 (34.03–82.84) E_max_ = 61%	10.45 (1.43–76.18) E_max_ = 54.4%	>100
CCRF-CEM	0.78 (0.25–2.37) E_max_ = 66.2%	25.18 (16.61–38.18) E_max_ = 69.2%	9.69 (4.39–21.39) E_max_ = 69.5%	>100

Data expressed from 3 independent tests (N3), with a 95% confidence interval which is presented between “()” when it was possible to determine the CC_50_. E_max_ represents the maximum effect of the compound at its maximum concentration used.

**Table 4:** Combination index (CI) determined from the combination of gedatolisib, LASSBio-1911, tubastatin A and LASSBio-2208 in CCRF-CEM using the MTT assay at 48 h of incubation.

Combinations: X×CC_50_ (A) + X×CC_50_ (B)	Gedatolisib (A) + LASSBio-1911 (B)	Gedatolisib (A) + Tubastatin A (B)	Gedatolisib (A) + LASSBio-2208 (B)	LASSBio-2208 (A) + LASSBio-1911 (B)	Tubastatin A (A) + LASSBio-2208 (B)
X = 0.25	Moderate antagonism (CI = 1.25)	Nearly additive (CI = 1.05)	Moderate antagonism (CI = 1.24)	Moderate antagonism (CI = 1.23)	Slight synergism (CI = 0.86)
X = 0.5	Moderate antagonism (CI =1.27)	Moderate antagonism (CI =1.32)	Slight synergism (CI = 0.89)	Moderate synergism (CI = 0.81)	Antagonism (CI =1.53)
X = 1	Nearly additive (CI = 0.96)	Synergism (CI = 0.48)	Synergism (CI = 0.63)	Synergism (CI = 0.56)	Synergism (CI = 0.38)
X = 2	Synergism (CI = 0.53)	Synergism (CI = 0.51)	Synergism (CI = 0.33)	Synergism (CI = 0.61)	Synergism (CI = 0.48)
X = 4	Moderate synergism (CI = 0.77)	Nearly additive (CI = 1.01)	Strong synergism (CI = 0.15)	Nearly additive (CI = 0.96)	Nearly additive (CI = 0.97)

Data expressed from 3 independent tests (*n* = 3). CI: combination index. (A) and (B) are the compounds that were combined in “X×CC_50_” proportion.

**Table 5:** Combination index (CI) determined for the combination of the compounds gedatolisib, LASSBio-1911, tubastatin A and LASSBio-2208 in the immortalized MOLT-4 cell lines using the MTT assay (48 h incubation).

Combinations: X×CC_50_ (A) + X×CC_50_ (B)	Gedatolisib (A) + LASSBio-1911 (B)	Gedatolisib (A) + Tubastatin A (B)	Gedatolisib (A) + LASSBio-2208 (B)	LASSBio-2208 (A) + LASSBio-1911 (B)	Tubastatin A (A) + LASSBio-2208 (B)
X = 0.25	Moderate antagonism (CI = 1.21)	Moderate synergism (CI = 0.72)	Moderate synergism (CI = 0.77)	Moderate antagonism (CI = 1.28)	Strong antagonism (CI = 3.69)
X = 0.5	Nearly additive (CI = 0.91)	Synergism (CI = 0.69)	Slight synergism (CI = 0.65)	Slight antagonism (CI = 1.18)	Nearly additive (CI =1.02)
X = 1	Synergism (CI = 0.49)	Synergism (CI = 0.41)	Synergism (CI = 0.44)	Synergism (CI = 0.54)	Synergism (CI = 0.53)
X = 2	Synergism (CI = 0.54)	Moderate synergism (CI = 0.80)	Synergism (CI = 0.34)	Synergism (CI = 0.64)	Slight synergism (CI = 0.87)
X = 4	Nearly additive (CI = 1.03)	Antagonism (CI = 1.59)	Synergism (CI = 0.47)	Moderate antagonism (CI = 1.31)	Antagonism (CI = 1.86)

Data expressed from 3 independent tests (*n* = 3). CI: combination index. (A) and (B) are the compounds that were combined in “X×CC_50_” proportion.

**Table 6:** Parallel artificial membrane permeability assay (PAMPA) for blood–brain barrier (BBB) and gastrointestinal tract (GIT).

Compounds	Pe. Exp. BBB (10^−6^ cm/s)	Classification BBB	Pe. Exp. GIT (10^−6^ cm/s)	Fraction Absorbed (%)	Classification GIT
LASSBio-2208	0.69	CNS−	0.39	13.78	Low
LASSBio-1911	0.29	CNS−	0.592	20.15	Low
Gedatolisib	Insoluble		1.74	48.40	Medium
Tubastatin A	5.54	CNS+	1.982	52.93	Medium

**Table 7:** Pharmacokinetic parameters calculated for the plasma stability assay to LASSBio-2208 and LASSBio-1911.

Compounds	Metabolization Rate (%)	Elimination Rate Constant (*k*)	t_1/2_ (min)	Recovery (%)
LASSBio-2208	20.61	0.0008	866.25	75.47
LASSBio-1911	36.14	0.0018	385.00	73.36

**Table 8:** Determination of the unbound fraction (*ƒ_u_*_(mic)_) in the nonspecific microsomal binding.

Compounds	Percentage of *ƒ_u_*_(mic)_
Tolbutamide *	1.113
LASSBio-2208	0.937
LASSBio-1911	0.004
Gedatolisib	0.503
Tubastatin A	0.131

* Literature value: 0.97 [31].

## Data Availability

Data are contained within the article.

## Supplementary Materials

The following supporting information can be downloaded at: https://www.mdpi.com/article/10.3390/ph17030389/s1, Figures S1–S4: Purity analysis of gedatolisb, tubastatin A, LASSBio-2208 and LASSBio-1911 by HPLC/UV.
