# Supplementary material for: Cytotoxic and Antiproliferative Activity of LASSBio-2208 and the Attempts to Determine Its Drug Metabolism and Pharmacokinetics In Vitro Profile
Source: Pharmaceuticals (Basel). 2024 Mar 18;17(3):389. doi: 10.3390/ph17030389 (PMC10974272; doi:10.3390/ph17030389)
Supplement: Supplementary file 1 [file pharmaceuticals-17-00389-s001.zip › pharmaceuticals-2894074-supplementary.pdf]

Cytotoxic and antiproliferative activity of LASSBio-2208 and the attempts to determine its drug metabolism and pharmacokinetics in vitro profile.

Raysa Magali Pillpe-Meza[1,2], Wesley Leandro Gouveia[1,2], Gisele Barbosa[1], Carlos A. M. Fraga[1,2], Eliezer J. Barreiro[1,2], Lídia Moreira Lima[1,2*].

[1]Laboratório de Avaliação e Síntese de Substâncias Bioativas (LASSBio®), Instituto Nacional de Ciência e Tecnologia de Fármacos e Medicamentos (INCT-INOFAR), Universidade Federal do Rio de Janeiro, Rio de Janeiro 21941-902, RJ, Brazil; rpillpe@gmail.com (R.M.P-M.); bgiselejf@gmail.com (G.B.); wleandrogouveia@gmail.com (W.L.G.); cmfraga@ccsdecania.ufrj.br (C.A.M.F.); ejbarreiro@ccsdecania.ufrj.br (E.J.B.)

[2]Programa de Pós-Graduação em Farmacologia e Química Medicinal, Instituto de Ciências Biomédicas, Universidade Federal do Rio de Janeiro, Rio de Janeiro 21941-902, RJ, Brazil

*Correspondence: lmlima23@gmail.com or lidialima@ufrj.br

Table of contents

LC/UV Analyisis.. 2

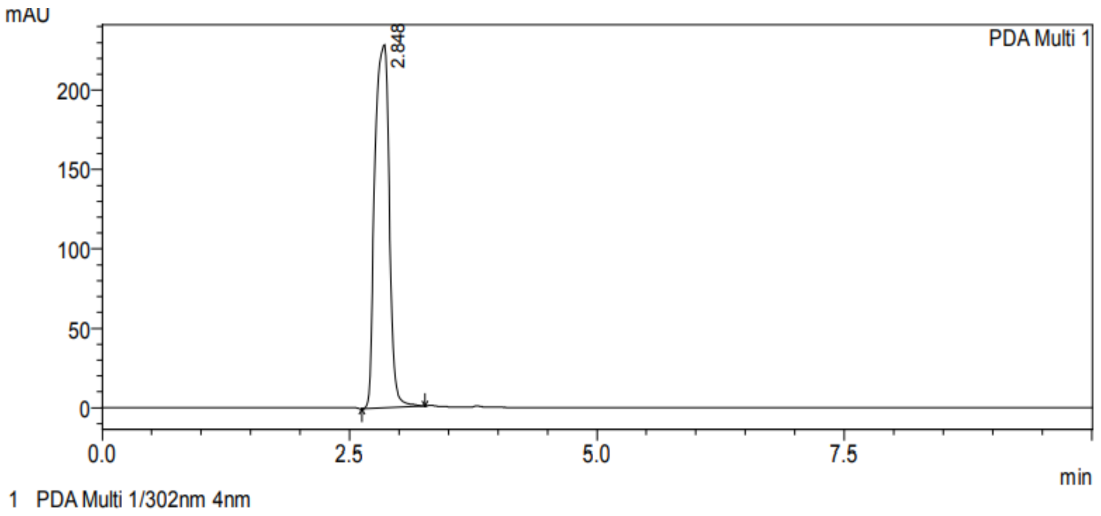

PeakTable

PDA Ch1 302nm 4nm

Peak#	Ret. Time	Area	Height	Area %	Height %
1	2.848	2320143	228571	100.000	100.000
Total		2320143	228571	100.000	100.000

Figure S1 – Purity analysis of the Gedatolisib standard by HPLC. Samples analyzed at 302 nm. Isocratic mobile phase acetonitrile - acidified water (9:1) in 10 min at a flow rate of 1mL/min.

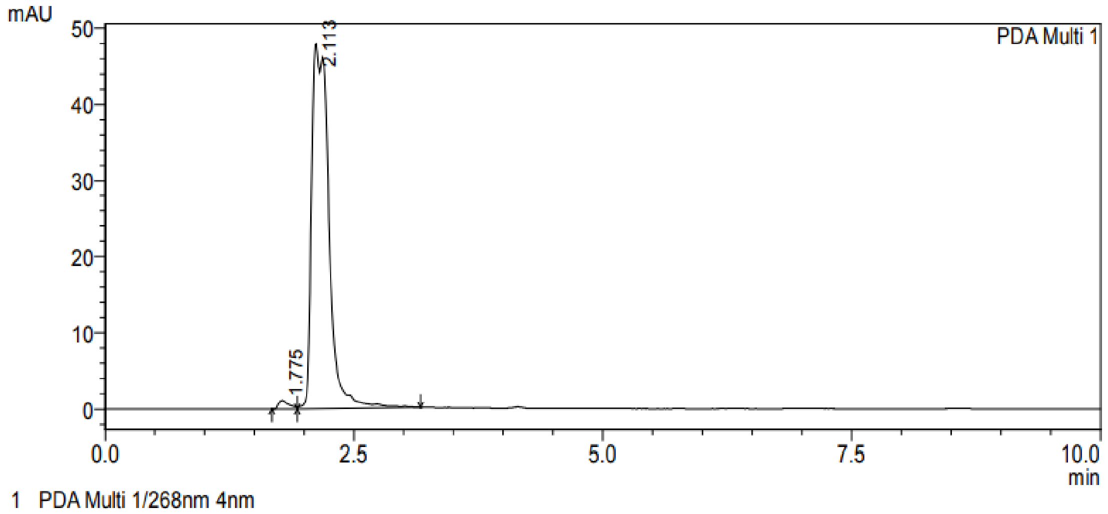

PeakTable

PDA Ch1 268nm 4nm

Peak#	Ret. Time	Area	Height	Area %	Height %
1	1.775	7949	1072	1.372	2.192
2	2.113	571440	47830	98.628	97.808
Total		579389	48902	100.000	100.000

Figure S2 – Purity analysis of the Tubastatin A standard by HPLC. Samples analyzed at 268 nm. Isocratic mobile phase acetonitrile-acidified water (5:5) in 10 min at a flow rate of 1mL/min.

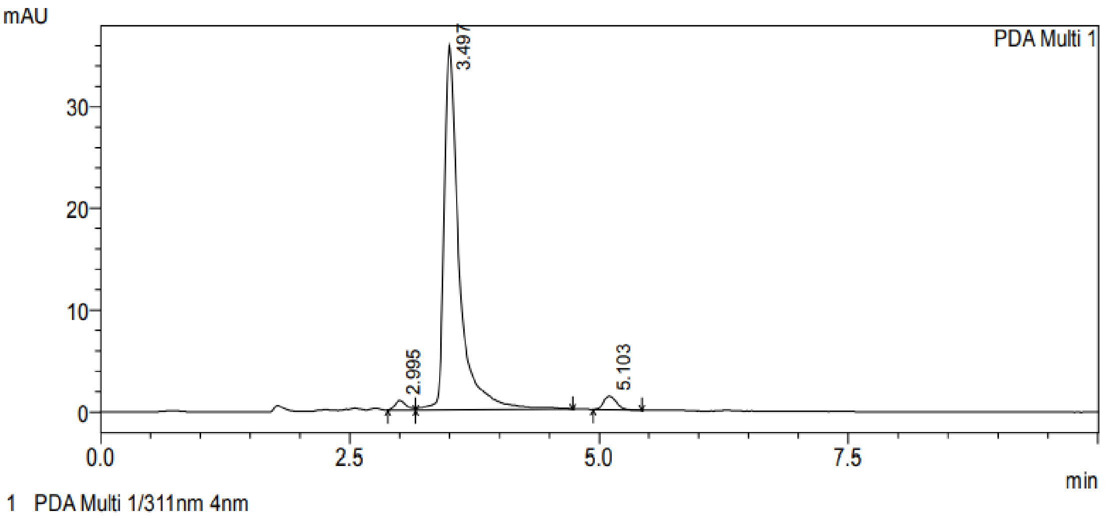

PeakTable

PDA Ch1 311nm 4nm

Peak#	Ret. Time	Area	Height	Area %	Height %
1	2.995	7632	943	1.922	2.481
2	3.497	377472	35726	95.052	93.973
3	5.103	12016	1348	3.026	3.546
Total		397120	38018	100.000	100.000

Figure S3 – Purity analysis of the compound LASSBio-2208 by HPLC. Samples analyzed at 311 nm. Isocratic mobile phase acetonitrile-acidified water (5:5) in 10 min at a flow rate of 1mL/min.

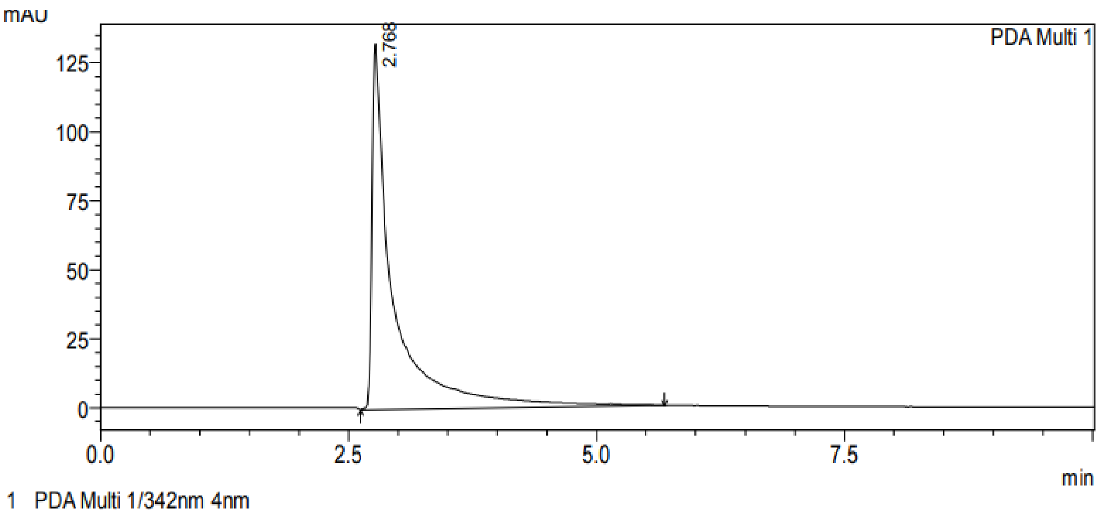

PeakTable

PDA Ch1 342nm 4nm

Peak#	Ret. Time	Area	Height	Area %	Height %
1	2.768	1968344	132432	100.000	100.000
Total		1968344	132432	100.000	100.000

Figure S4 – Purity analysis of the compound LASSBio-1911 by HPLC. Samples analyzed at 347 nm. Isocratic mobile phase acetonitrile - acidified water (9:1) in 10 min at a flow rate of 1mL/min.
